# Does the Choice of Extraction Site During Minimally Invasive Colorectal Surgery Change the Incidence of Incisional Hernia? Protocol for a Systematic Review and Network Meta-Analysis

**DOI:** 10.29337/ijsp.164

**Published:** 2021-09-20

**Authors:** Jeremy Meyer, Constantinos Simillis, Heman Joshi, Athanasios Xanthis, James Ashcroft, Nicolas Buchs, Frédéric Ris, R. Justin Davies

**Affiliations:** 1 Cambridge Colorectal Unit, Addenbrooke’s Hospital, Cambridge University Hospitals NHS Foundation Trust, Hills Road, Cambridge CB2 0QQ, GB; 2 Division of Digestive Surgery, University Hospitals of Geneva, Rue Gabrielle-Perret-Gentil 4, 1211 Genève 14, CH; 3 Medical School, University of Geneva, Rue Michel-Servet 1, 1205 Genève, CH

**Keywords:** Incisional hernia, colorectal surgery, extraction, specimen, hernia

## Abstract

**Background:**

Various sites are used for specimen extraction in oncological minimally invasive colorectal surgery. The objective is to determine if the choice of extraction site modulates the incidence of incisional hernia (IH).

**Methods/design:**

A systematic review will be performed in accordance to the Preferred Reporting Items for Systematic Reviews and Meta-Analyses (PRISMA) statement. MEDLINE, Embase and CENTRAL will be searched to look for original studies reporting the incidence of IH after minimally invasive colorectal surgery. Studies will be excluded from the analysis if: 1) they do not report original data, 2) the outcome of interest (incidence of incisional hernia) is not clearly reported and does not allow to extrapolate and/or calculate the required data for network meta-analysis, 3) they include pediatric patients, 4) they include a patients’ population with a conversion rate to laparotomy >10%, 5) they do not compare at least two different extraction sites for the operative specimen, 6) they report patients who underwent pure (and not hybrid) natural orifice transluminal endoscopic surgery (NOTES). Network meta-analysis will be performed to determine the incidence of IH per extraction site.

**Discussion:**

By determining which specimen extraction site leads to reduced rate of IH, this systematic review and network meta-analysis will help colorectal surgeons to choose their extraction site and reduce the morbidity and costs associated with IH.

**Registration:**

The systematic review and meta-analysis protocol is registered in the International Prospective Register of Ongoing Systematic Reviews (PROSPERO) with number CRD42021272226.

**Highlights:**

- Various sites are used for specimen extraction in oncological minimally invasive colorectal surgery, and the choice of the site may probably modulate the incidence of incisional hernia.
- The present protocol aims to design a systematic review which will identify original studies comparing two extraction sites during minimally invasive colorectal surgery in terms of incidence of incisional hernia.
- Network meta-analysis will be performed to determine the incidence of IH per extraction site.

## Background

Incisional hernia (IH) occurs in 3.8% of patients within 5 years of a laparotomy, an incidence which raises up to 7.7% after colorectal surgery procedures [1]. IH significantly impairs quality of life [2], is associated with an important morbidity as it notably exposes the patients to small bowel obstruction, and carries a significant economic burden for healthcare systems. For instance, analysis of the Nationwide Inpatient Sample revealed that 72.1% of all ventral hernia repairs in the USA are IH repairs [3]. The cost for one IH repair is estimated to be of 15,899 USD (95% CI $15,394-$16,404) in the USA [4], and 10,107 euros in France when including indirect costs [5]. Moreover, IH have a 10-year cumulative recurrence rate of 63% after suture repair and of 32% after mesh repair [6].

Widespread adoption of minimally invasive techniques for colorectal surgery unfortunately has not lead to the expected decrease in the incidence of IH, and publications in the field have reported mixed results [7,8,9]. This might be explained by the necessity to sometimes proceed to conversion to open surgery [9], but also by the extraction of the operative specimen which requires an incision of sufficient length, especially in oncology cases. Moreover, depending on the surgeon’s preferences, the patient’s anatomy and the complexity of the surgical procedure, this incision can be used to perform manual assistance during the dissection of the tissues, extra-corporeal mesentery/vessel division, extra-corporeal bowel division and/or extra-corporeal anastomosis. These considerations usually dictate the preferred site for specimen extraction and its length.

Of interest, midline incision [10,11] and length of incision [11] were identified as risk factors for the development of IH after laparotomy. In laparoscopic colorectal surgery, midline incisions were also identified as being at higher risk for IH than off-midline incisions. For instance, the pooled incidence of IH was reported to be 10.6% if the specimen was extracted through the midline, versus 3.7% for transverse incision and 0.9% for Pfannenstiel incision [12].

Considering the diversity in potential extraction sites, including the midline, Pfannenstiel incision, left iliac fossa transverse incision, right iliac fossa transverse incision, McBurney’s incision, transanal natural orifice specimen extraction (NOSE), transvaginal NOSE and stoma site, a network meta-analysis would allow determination of which incision for specimen extraction should be preferred to avoid IH in colorectal surgery.

The objective of the present study is to determine if the incidence of IH after laparoscopic colorectal surgery depends on the chosen specimen extraction site (Table S1).

## Methods and analysis

### Literature search

The systematic review will be performed according to the Preferred Reporting Items for Systematic Reviews and Meta-Analyses (PRISMA) [13]. MEDLINE and Embase will be searched from inception to the 1^st^ of August 2021 for observational and interventional studies written in English including patients who benefited from minimally invasive colorectal surgery and comparing at least two specimen extraction sites in terms of incidence of IH. Review of titles and abstracts from studies in the field identified by experts or included into existing reviews [12,14] allowed to develop a literature search strategy, which is reported in Table S2. Additional records will be identified by manual search of the reference lists of the included publications and existing reviews.

### Inclusion and exclusion criteria

Case series, conference abstracts, letters to the editor and secondary analyses of previously published papers will be excluded. Studies including pediatric population and/or a patients’ population with a conversion rate to laparotomy >10%, studies not comparing two different extraction sites for the operative specimen, studies about pure (and not hybrid) natural orifice transluminal endoscopic surgery (NOTES) will also be excluded. Of note, studies reporting on minimally invasive surgery associated with NOSE and/or on single incision laparoscopic surgery (SILS) will be included. Eligible studies will be independently screened for inclusion using the Covidence software [15] by two authors (JM, JA). Discrepancies will be solved by a third author (AX).

### Data extraction

The following variables will be extracted by two authors (JM, JA) from included publications: first author, publication year, country of investigation, study period, study design, number of included patients, number of males, mean/median age of patients, types(s) of colorectal procedure(s) performed, number of patients with stoma, types of extraction sites used for specimen extraction, number of patients per group (per site of specimen extraction), duration of mean/median follow-up for the occurrence of IH, definition of IH, reported site(s) of IH, methods for assessing the occurrence of IH, timepoint(s) used for detection of potential IH, and incidence of IH per group (per site of specimen extraction).

### Statistical analysis

Stata/IC 11 (StataCorp LP, College Station, Texas, USA) will be used to draw a network plot summarizing comparisons between the different extraction sites used for specimen extraction (midline incision, Pfannenstiel incision, left iliac fossa transverse incision, right iliac fossa transverse incision, McBurney incision, transanal NOSE, transvaginal NOSE and stoma site) in terms of incidence of IH (CS). Any treatment not connected to the other treatments through the network plot will be excluded from the analysis of the incidence of IH. A Bayesian network meta-analysis will be conducted using the Markov chain Monta Carlo method in WinBUGS 1.4 (MRC Biostatistics Unit, Cambridge, and Imperial College School of Medicine, London, UK). For binary data, a binomial model will be used for the analysis, and the odds ratio (OR) will be calculated. The treatment contrast for any two treatments will be modelled as a function of comparisons between each individual treatment and an arbitrarily selected reference group. The probability of ranking of a treatment (i.e. that a treatment ranks as the best treatment, second best treatment, etc.) for each outcome of interest will be calculated. If the pooled sample size will allow it, subgroups analyses will be performed subdividing the pooled patients’ populations according to the type of surgical procedure (right colectomy versus left colectomy versus anterior resection of the rectum), the methods to assess the presence or not of IH (CT versus clinical examination versus mixed), and the type of IH (all IH versus IH at site of specimen extraction), and the methodological quality of included studies.

### Quality assessment

Two authors (JM, JA) will perform the critical appraisal of the included studies. Risk of bias will be assessed using the Newcastle-Ottawa scale for observational studies, and the Cochrane Collaboration’s tool for assessing risk of bias in randomized trials for randomized trials [16]. Discrepancies will be solved by a third author (AX). Studies will be ranked as at low risk of bias, at moderate risk of bias or at high risk of bias for the studied outcome (incidence of IH).

### Registration

The systematic review and network meta-analysis protocol is registered in the International Prospective Register of Ongoing Systematic Reviews (PROSPERO) with number CRD42021272226.

## Discussion

This systematic review and network meta-analysis will allow determining which specimen extraction site should be favoured by colorectal surgeons to reduce the incidence of IH. This is of importance to reduce patients’ morbidity and costs for healthcare. Depending on the results obtained, recommendations could be made for colorectal surgeons to favor one extraction site over another. The main strength of this meta-analysis will be to be the first one in the field to compare several specimen extraction sites using network meta-analytic tools. Its main limitations will be to not be limited to RCTs (and to also pool observational studies), and potential heterogeneity among included studies in terms of definition and detection of IH, and in terms of patients’ populations. Also, lengths of incisions will not be assessed.

## Additional Files

The additional files for this article can be found as follows:

10.29337/ijsp.164.s1 Table S1. PICOS table.

10.29337/ijsp.164.s2 Table S2. Literature search strategy.
