# Supplementary material for: Does the Choice of Extraction Site During Minimally Invasive Colorectal Surgery Change the Incidence of Incisional Hernia? Protocol for a Systematic Review and Network Meta-Analysis
Source: Int J Surg Protoc. 2021 Sep 20;25(1):216–9. doi: 10.29337/ijsp.164 (PMC8462477; doi:10.29337/ijsp.164)
Supplement: Table S1. — PICOS table. [file ijsp-25-1-164-s1.pdf]

Database	Search build
MEDLINE (Pubmed)	(“Extraction”[Title/Abstract] OR “Retrieval”[Title/Abstract] OR “Specimen”[Title/Abstract])<br>AND<br>("incisional hernia"[MeSH Terms] OR “incisional hernia”[Title/Abstract])<br>AND<br>(“laparoscop*”[Title/Abstract] OR "laparoscopy"[MeSH Terms] OR “robotic”[Title/Abstract] OR “robotic”[MeSH Terms] OR “minimally invasive surgery”[MeSH Terms])<br>AND<br>("colorectal cancer"[MeSH Terms] OR “colon”[Title/Abstract] OR “colonic”[Title/Abstract] OR “anterior resection”[Title/Abstract] OR “colectomy”[Title/Abstract] OR “resection”[Title/Abstract] OR “rectal cancer”[Title/Abstract] OR “colon cancer”[Title/Abstract])
EMBASE (OvidSP)	1. extraction:ab,ti<br>2. 'incisional hernia'/exp/mj<br>3. 'colorectal surgery'/exp/mj<br>1, 2 AND 3
Cochrane Central Register of Controlled Trials (CENTRAL)	#1 extraction:ab,ti<br>#2 'incisional hernia'/exp/mj<br>#3 'colorectal surgery'/exp/mj<br>#4 (#1 AND #2 AND #3)
Other sources	Screening of references of included articles and of existing reviews in the field

Table S1. PICOS table
