# Supplementary material for: Does the Choice of Extraction Site During Minimally Invasive Colorectal Surgery Change the Incidence of Incisional Hernia? Protocol for a Systematic Review and Network Meta-Analysis
Source: Int J Surg Protoc. 2021 Sep 20;25(1):216–9. doi: 10.29337/ijsp.164 (PMC8462477; doi:10.29337/ijsp.164)
Supplement: Table S2. — Literature search strategy. [file ijsp-25-1-164-s2.pdf]

Questions	Answers
P - Population	Minimally invasive colorectal surgery
I - Intervention	Extraction site of the operative specimen
C - Control	Alternative extraction site of the operative specimen
O - Outcome	Incidence of incisional hernia

Table S2. Literature search strategy
